# LKB1 Haploinsufficiency Cooperates With *Kras* to Promote Pancreatic Cancer Through Suppression of p21-Dependent Growth Arrest

**DOI:** 10.1053/j.gastro.2010.04.055

**Published:** 2010-08

**Authors:** Jennifer P. Morton, Nigel B. Jamieson, Saadia A. Karim, Dimitris Athineos, Rachel A. Ridgway, Colin Nixon, Colin J. McKay, Ross Carter, Valerie G. Brunton, Margaret C. Frame, Alan Ashworth, Karin A. Oien, T.R. Jeffry Evans, Owen J. Sansom

**Affiliations:** ⁎ Beatson Institute for Cancer Research, Garscube Estate, Glasgow, UK; ‡ Centre for Oncology and Applied Pharmacology, Division of Cancer Sciences and Molecular Pathology, University of Glasgow, Glasgow, UK; § West of Scotland Pancreatic Unit, Glasgow Royal Infirmary, Alexandra Parade, Glasgow, UK; ∥ Edinburgh Cancer Research Centre, Institute of Genetics and Molecular Medicine, University of Edinburgh, Edinburgh, UK; ¶ The Breakthrough Breast Cancer Research Centre, The Institute of Cancer Research, London, UK

**Keywords:** Pancreatic Cancer, Kras, LKb1, p21, AMPK, adenosine monophosphate–activated protein kinase, PanIN, pancreatic intraepithelial neoplasia, PDAC, pancreatic ductal adenocarcinoma.

## Abstract

**Background & Aims:**

Patients carrying germline mutations of *LKB1* have an increased risk of pancreatic cancer; however, it is unclear whether down-regulation of *LKB1* is an important event in sporadic pancreatic cancer. In this study, we aimed to investigate the impact of LKB1 down-regulation for pancreatic cancer in mouse and human and to elucidate the mechanism by which Lkb1 deregulation contributes to this disease.

**Methods:**

We first investigated the consequences of Lkb1 deficiency in a genetically modified mouse model of pancreatic cancer, both in terms of disease progression and at the molecular level. To test the relevance of our findings to human pancreatic cancer, we investigated levels of LKB1 and its potential targets in human pancreatic cancer.

**Results:**

We definitively show that *Lkb1* haploinsufficiency can cooperate with oncogenic *Kras*^*G12D*^ to cause pancreatic ductal adenocarcinoma (PDAC) in the mouse. Mechanistically, this was associated with decreased p53/p21-dependent growth arrest. Haploinsufficiency for p21 (*Cdkn1a)* also synergizes with *Kras*^*G12D*^ to drive PDAC in the mouse. We also found that levels of LKB1 expression were decreased in around 20% of human PDAC and significantly correlated with low levels of p21 and a poor prognosis. Remarkably, all tumors that had low levels of LKB1 had low levels of p21, and these tumors did not express mutant p53.

**Conclusions:**

We have identified a novel LKB1-p21 axis that suppresses PDAC following *Kras* mutation in vivo. Down-regulation of LKB1 may therefore serve as an alternative to p53 mutation to drive pancreatic cancer in vivo.

Pancreatic cancer is the fourth most common cause of cancer deaths worldwide, with an estimated 5-year overall survival of <5%.^1^ The highly aggressive nature of this disease, combined with the anatomical location of tumors, results in 90% of patients having surgically unresectable disease at the time of diagnosis.^2^ The pancreas consists of 3 main cell types—islet cells, acinar cells, and duct cells. Tumors can arise from any of these cell types, but approximately 90% of cases are pancreatic ductal adenocarcinoma (PDAC). PDAC arises from precursor lesions called pancreatic intraepithelial neoplasms (PanINs).^3^ The formation of PanIN lesions and the progression to invasive adenocarcinoma are driven by activation of the *KRAS* oncogene in about 90% of cases,^4^ accompanied by loss of function of tumor suppressors, most commonly the Ink4a, p53, and Smad4 tumor suppressors.^3^

Certain inherited genetic lesions have also been shown to confer a predisposition to pancreatic cancer. Mutations in the *LKB/STK11* tumor suppressor gene result in the Peutz–Jeghers syndrome,^5,6^ an autosomal-dominant condition characterized by hamartomatous polyps of the gastrointestinal tract and a dramatically increased risk of epithelial malignancies at other sites, including a >100-fold increased risk of pancreatic cancer.^7-9^ Restoration of silenced *LKB1* in human pancreatic carcinoma cells induces apoptosis in vitro.^10^ Furthermore, *LKB1* gene inactivation has been observed in intraductal papillary mucinous neoplasms of the pancreas.^11^

*Lkb1* knockout mice are not viable, and embryos survive only until embryonic day E9.5 because of neural tube defects and vascular abnormalities.^12^ However, *Lkb1*^+/−^ mice are viable and mirror human Peutz–Jeghers syndrome in that they develop benign intestinal polyps (hamartomas) and have an increased risk of a range of cancers later in life.^13-16^ However, the consequences of Lkb1 deficiency in the pancreas have not been well-studied thus far, and the mechanisms by which its loss may contribute to pancreatic cancer are unknown.

*Lkb1* encodes a serine/threonine kinase that activates a number of downstream kinases, including the adenosine monophosphate−activated protein kinase (AMPK), which responds to energy stress by negatively regulating the mammalian target of rapamycin kinase.^17^ Lkb1 is also able to regulate cell growth and apoptosis, potentially through interaction with the tumor suppressor p53.^18^ Ectopic expression of Lkb1 in cells lacking the endogenous protein induces p21 expression and cell-cycle arrest in a p53-dependent manner, and Chromatin Immunoprecipitation analysis has revealed that Lkb1 is recruited to the *p21* promoter by p53.^19-21^ Lkb1 deficiency has also been shown to prevent culture-induced senescence, although paradoxically it renders cells resistant to subsequent transformation by Ha-Ras.^13^

Using cre-lox technology to target endogenous expression of Kras^G12D^ to the mouse pancreas through the *Pdx1* pancreatic progenitor cell gene promoter results in formation of PanINs.^22^ However, these lesions fail to rapidly progress and only develop into invasive pancreatic adenocarcinoma at low frequency unless additional genetic lesions are introduced. In this study, we have assessed whether Lkb1 loss can promote tumorigenesis in this model and found a dramatic acceleration of tumorigenesis in mice carrying a single conditional knockout allele of Lkb1. We have also demonstrated that this is associated with decreased p21-dependent growth arrest.

## Materials and Methods

### Genetically Modified Mice and Animal Care

The *Pdx1-Cre*, *LSL-Kras*^*G12D*^, *Lkb1*^*flox/flox*^, and *Cdkn1a*^*−/−*^ mice have been described previously.^23-26^ For further information, see Supplementary Materials.

### Immunohistochemistry

Immunohistochemical analysis was performed on formalin-fixed paraffin-embedded sections according to standard protocols. For detailed protocols, see Supplementary Materials.

### Senescence-Associated β-Galactosidase Staining

We stained cryosections of mouse pancreas or tumor for senescence-associated β-galactosidase activity according to manufacturer's protocol (Cell Signaling Technology, Danvers, MA) and counterstained them with nuclear fast red solution.

### Laser Capture Microdissection and RNA Isolation

Frozen tissue was sectioned (at 15–20 μm) onto PALM-PEN membrane slides and lightly stained with hematoxylin. Laser capture microdissection was performed using the P.A.L.M. MicroLaser System. RNA was isolated with the RNA easy extraction kit (Qiagen, Hilden, Germany).

### Reverse-Transcription Polymerase Chain Reaction

Total RNA was reverse transcribed to complementary DNA using the Superscript III kit (Invitrogen, Carlsbad, CA) according to manufacturer's instructions. For further information and primers, see Supplementary Materials.

### Immunoblotting

Tissue samples were homogenized into supplemented Tissue Protein Extraction Reagent (Thermo Scientific, Waltham, MA) in the Precellys 24 (Stretton Scientific, Stretton, UK). Lysates were resolved by 10% Bis-Tris gel electrophoresis (Invitrogen). Proteins were transferred to polyvinylidene difluoride membrane, blocked, and probed with antibodies against Lkb1 (Cell Signaling Technology) 1:1000; phospho-AMPK (Cell Signaling Technology) 1:1000, and β-tubulin (Sigma) 1:5000.

### Tissue Microarray Analysis

The human pancreatico-biliary tissue microarray was created within the West of Scotland Pancreatic Unit, University Department of Surgery, Glasgow Royal Infirmary. For further information, see Supplementary Materials.

## Results

### Lkb1 Heterozygosity Accelerates *Kras^G12D^*-Induced Pancreatic Cancer

**Figure 1:**
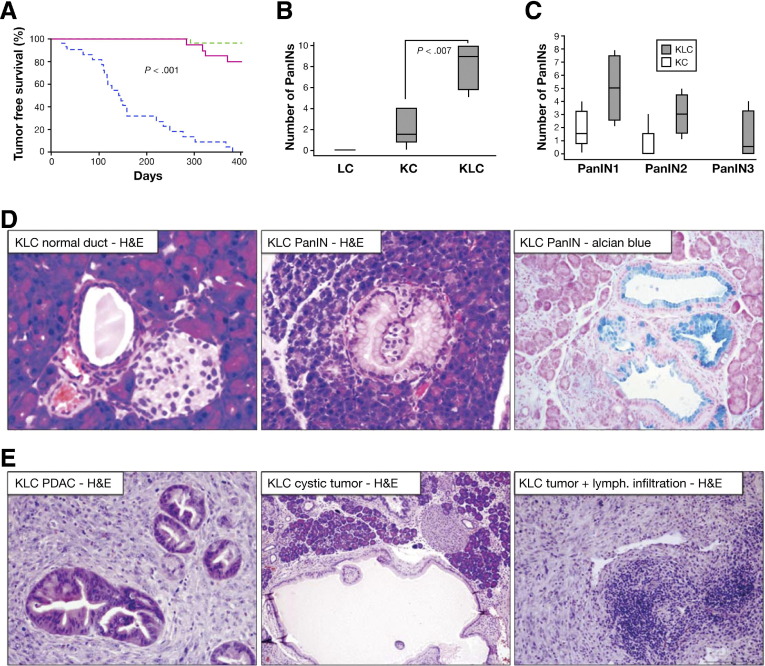
*Lkb1* heterozygosity combined with Kras^G12D^ is sufficient to induce pancreatic cancer. (*A*) Kaplan–Meier survival curve showing tumor-free survival of *Pdx1-Cre Lkb1*^*flox/+*^ (LC, *green dashed line*), *Pdx1-Cre Kras*^*G12D/+*^ (KC, *red solid line*), and *Pdx1-Cre Kras*^*G12D/+*^*Lkb1*^*flox/+*^ (KLC, *blue dashed line*) mice. (*B*) *Boxplot* showing number of pancreatic intraepithelial neoplasia (PanINs) per histopathological section of pancreas from wild-type, KC, and KLC mice (n = 6 mice per genotype) as indicated (*P* = .007). (*C*) *Boxplot* showing number of PanINs of grades 1−3 per histopathological section of pancreas from wild-type, KC, and KLC mice (n = 6 mice per genotype) as indicated. (*D*) H&E-stained sections of a normal duct, a PanIN lesion, and an Alcian blue−stained section of a PanIN lesion arising in the pancreas of a 6-week-old KLC mouse, as indicated. (*E*) H&E-stained sections of pancreatic ductal adenocarcinoma (PDAC) arising in KLC mice, with some tumors exhibiting a cystic component and others exhibiting increased lymphocytic involvement.

To determine whether Lkb1 deficiency could act in synergy with activated Kras signaling to promote pancreatic tumorigenesis, we crossed *Lkb1*^*flox/+*^ mice to *Pdx1-Cre, Kras*^*G12D/+*^ mice to generate cohorts of *Pdx1-Cre, Kras*^*G12D/+*^ (KC), *Pdx1-Cre, Lkb1*^*flox/+*^ (LC), and *Pdx1-Cre, Kras*^*G12D/+*^*, Lkb1*^*flox/+*^ (KLC) mice.^25^ These mice were monitored for pancreatic tumor development and sacrificed at intervals, or as they showed signs of disease. Postmortem analysis was performed and tumors were diagnosed on the basis of gross pathology. The median time to PDAC of *Pdx1-Cre, Kras*^*G12D/+*^*, Lkb1*^*flox/+*^ (KLC) mice was just 141 days (n = 20, Kaplan–Meier curve, Figure 1*A*). As reported previously, *Pdx1-Cre, Kras*^*G12D/+*^ (KC) mice developed PanIN lesions within the pancreas that rarely progressed to adenocarcinoma within the 18-month duration of our experiment (n = 20).^22^*Pdx1-Cre, Lkb1*^*flox/+*^ (LC) mice remained disease-free for the duration of the experiment (n = 20). A small subset of the *Pdx1-Cre, Kras*^*G12D/+*^*, Lkb1*^*flox/+*^ (KLC) mice exhibited Cre-mediated recombination in their cecum, leading to intussusceptions, with a median onset of 47 days. These are not included in the Kaplan–Meier curve (Figure 1*A*).

This increased pancreatic cancer predisposition was not limited to invasive tumors; the number of PanINs observed in 6-week-old *Pdx1-Cre, Kras*^*G12D/+*^*, Lkb1*^*flox/+*^ (KLC) mice was significantly increased when compared with *Pdx1-Cre, Kras*^*G12D/+*^ (KC) mice (Figure 1*B*, *P* = .007). In addition, we also observed an increase in PanIN 2 and PanIN 3 lesions compared with KC mice (Figure 1*C*). Histological sections of tumors arising in *Pdx1-Cre, Kras*^*G12D/+*^*, Lkb1*^*flox/+*^ (KLC) animals were analyzed to ascertain the phenotype of these PanINs and tumors. PanIN lesions exhibited characteristic histologic changes of the normal duct, including expansion of the cytoplasm with associated mucin accumulation, which was confirmed by Alcian blue staining (Figure 1*D*, *right*), formation of papillary architecture, loss of polarity, appearance of atypical nuclei, and luminal budding (Figure 1*D*). A majority of *Pdx1-Cre, Kras*^*G12D/+*^*, Lkb1*^*flox/+*^ (KLC) tumors were PDAC (Figure 1*E*); however, some tumors exhibited a more cystic morphology (Figure 1*E*, *middle panel*), and enhanced immune cell infiltration was apparent in some tumors (Figure 1*E*, *right panel*), compared with the small number of tumors observed in older *Pdx1-Cre, Kras*^*G12D/+*^ (KC) mice. Our results show that Lkb1 deficiency can synergize with activated Kras to induce pancreatic tumor formation.

### Homozygous Loss of Lkb1 Is Sufficient to Initiate Pancreatic Tumorigenesis

**Figure 2:**
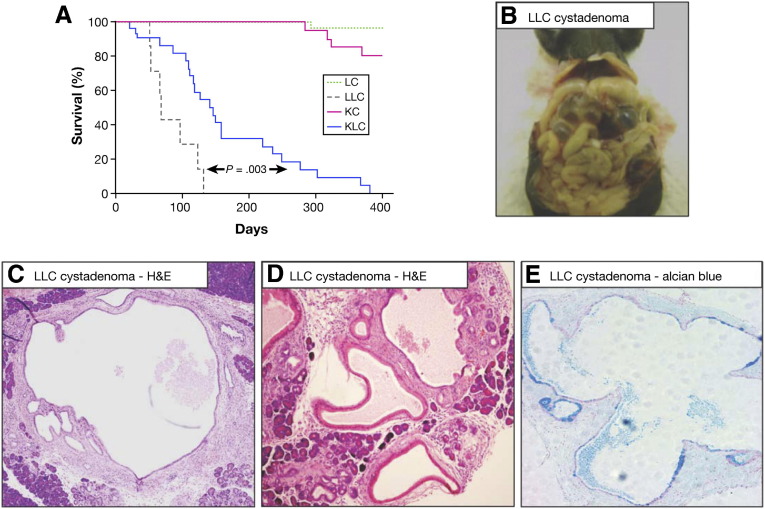
Homozygous loss of Lkb1 is sufficient to initiate pancreatic tumorigenesis. (*A*) Kaplan–Meier survival curve showing tumor-free survival of *Pdx1-Cre Lkb1*^*fl/+*^ mice (LC, *green dashed line*), *Pdx1-Cre Lkb1*^*fl/fl*^ mice (LLC, *blue dashed line*), *Pdx1-Cre Kras*^*G12D/+*^ mice (KC, *red solid line*), and *Pdx1-Cre Kras*^*G12D/+*^*Lkb1*^*fl/+*^ mice (KLC, *blue solid line*). (*B*) Gross pathology of a cystic pancreatic tumor arising in an LLC mouse. (*C*, *D*) H&E-stained sections of cystic pancreatic tumors arising in LLC mice. (*E*) Alcian blue−stained section of a cystic pancreatic tumor arising in an LLC mouse.

We also investigated whether loss of Lkb1 as a sole initiating genetic event was sufficient to induce pancreatic tumor formation in the mouse. We crossed *Lkb1*^*flox/+*^ mice to *Pdx1-Cre* mice and interbred the offspring to generate cohorts of *Pdx1-Cre Lkb1*^*flox/+*^ (LC) and *Pdx1-Cre Lkb1*^*flox/flox*^ (LLC) mice. We found that *Pdx1-Cre Lkb1*^*flox/flox*^ (LLC) mice develop pancreatic tumors with an incidence of 100% and a median survival of 68 days, while *Pdx1-Cre, Lkb1*^*flox/+*^ (LC) mice remained disease-free for 500 days (Figure 2*A*).*Pdx1-Cre Lkb1*^*flox/flox*^ (LLC) mice presented with abdominal distention, and tumors arising in these mice were mucinous cystadenomas characterized by the presence of multiple large cysts, in some cases at the expense of most of the normal pancreas tissue (Figure 2*B–E*). Tumors also exhibited excessive mucin production, as confirmed by Alcian blue staining (Figure 2*E*). We conclude that Lkb1 loss as a sole event is sufficient to initiate pancreatic tumor growth; however, those tumors are benign mucinous cystadenomas and Lkb1 loss alone is not sufficient to drive formation of PDAC. These results agree with a previous analysis of mice lacking Lkb1 specifically within the pancreas, in which mice developed pancreatic serous cystadenomas.^27^

### Lkb1 Haploinsufficiency Synergizes With Kras^G12D^ to Induce Pancreatic Cancer

**Figure 3:**
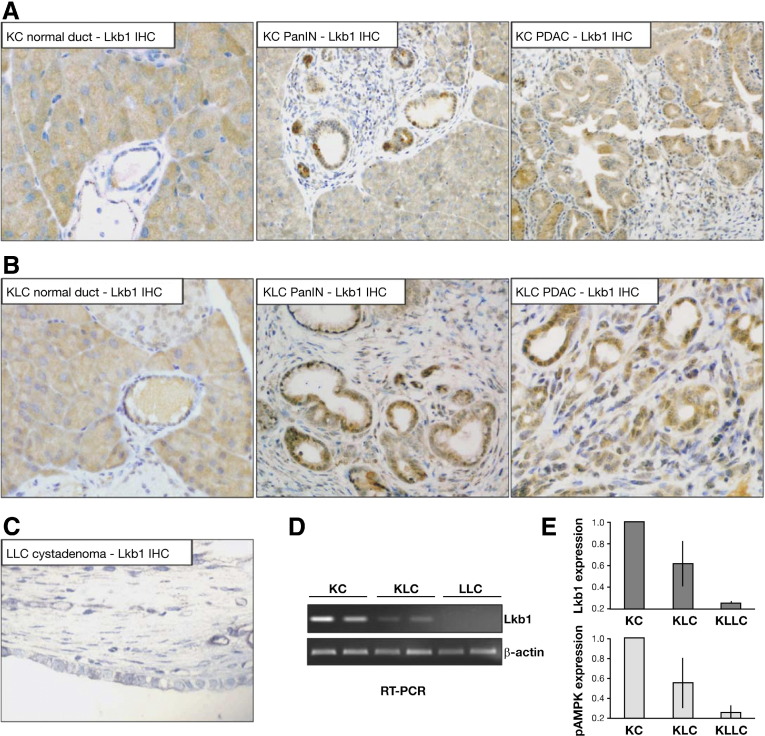
*Lkb1* haploinsufficiency synergizes with Kras^G12D^ to induce pancreatic cancer. (*A*) Immunohistochemical analysis of Lkb1 levels in normal duct, pancreatic intraepithelial neoplasia (PanIN), and pancreatic ductal adenocarcinoma (PDAC) arising in *Pdx1-Cre Kras*^*G12D/+*^ (KC) mice, as indicated. (*B*) Immunohistochemical analysis of Lkb1 levels in normal duct, PanIN, and PDAC arising in *Pdx1-Cre Kras*^*G12D/+*^*Lkb1*^*flox/+*^ (KLC) mice. (*C*) Immunohistochemical analysis of Lkb1 levels in cystadenoma arising in a *Pdx1-Cre Lkb1*^*flox/flox*^ (LLC) mouse. (*D*) Detection of the Lkb1 transcript by reverse-transcription polymerase chain reaction in tissue microdissected from lesions in frozen sections harvested from KC, KLC, and LLC mice. β-actin serves as control for RNA quantity and integrity. (*E*) Quantification of Western immunoblotting analysis of Lkb1 (*top panel*) and phospho−adenosine monophosphate−activated protein kinase (AMPK; *bottom panel*) protein levels in pancreatic tumor lysates from KC, KLC, and LLC mice. Levels were normalized against β-tubulin levels.

Because *Pdx1-Cre, Lkb1*^*flox/+*^ (LC) mice did not develop any disease, and *Pdx1-Cre Lkb1*^*flox/flox*^ (LLC) mice developed pancreatic tumors with a very short latency, we wondered whether the tumor phenotype we observed in *Pdx1-Cre, Kras*^*G12D/+*^*, Lkb1*^*flox/+*^ (KLC) mice was a result of complete loss of Lkb1 by loss of heterozygosity. Immunohistochemical analysis was performed to ascertain levels of Lkb1 in lesions and tumors from *Pdx1-Cre, Kras*^*G12D/+*^ (KC), and *Pdx1-Cre, Kras*^*G12D/+*^*, Lkb1*^*flox/+*^ (KLC) mice. Lkb1 was detected in the nucleus and cytoplasm of normal duct cells and *Pdx1-Cre, Kras*^*G12D/+*^ (KC) PanIN lesions and tumors as expected (Figure 3*A*),^28^ but also in lesions and tumors from *Pdx1-Cre, Kras*^*G12D/+*^*, Lkb1*^*flox/+*^ (KLC) mice (Figure 3*B*), indicating maintenance of the wild-type allele. As a control, Lkb1 immunohistochemistry was also carried out on tumors from *Pdx1-Cre Lkb1*^*flox/flox*^ (LLC) mice, and no Lkb1 was detected (Figure 3*C*).

**Supplementary Figure 1:**
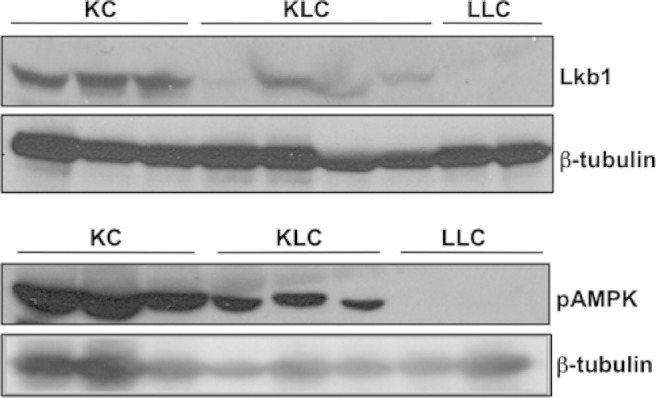
Western immunoblotting analysis of Lkb1 (*top panel*) and phospho−adenosine monophosphate−activated protein kinase (AMPK) (*bottom panel*) protein levels in pancreatic tumor lysates from KC, KLC, and LLC mice. Levels were normalized against β-tubulin levels.

Laser capture microdissection was also performed to isolate tissue from preneoplastic PanIN lesions and tumors arising in *Pdx1-Cre, Kras*^*G12D/+*^*, Lkb1*^*flox/+*^ (KLC) mice, and reverse-transcriptase polymerase chain reaction analysis showed transcription of wild-type Lkb1 in the resulting tumors (Figure 3*D*). Further, immunoblot analysis revealed only a decrease in Lkb1 levels, and a reduction in phosphorylation of the Lkb1 target, AMPK, in *Pdx1-Cre, Kras*^*G12D/+*^*, Lkb1*^*flox/+*^ (KLC) tumors, compared with the almost complete loss observed in tumors from *Pdx1-Cre Lkb1*^*flox/flox*^ (LLC) mice (Figure 3*E*, Supplementary Figure 1). These results demonstrate that Lkb1 is a haploinsufficient pancreatic tumor suppressor, and that lack of only 1 allele is sufficient, when combined with *Kras* mutation, to cause PDAC.

### Lkb1 Deficiency Limits Expression of the Tumor Suppressors p53 and p21 in PanIN Lesions

**Supplementary Figure 2:**
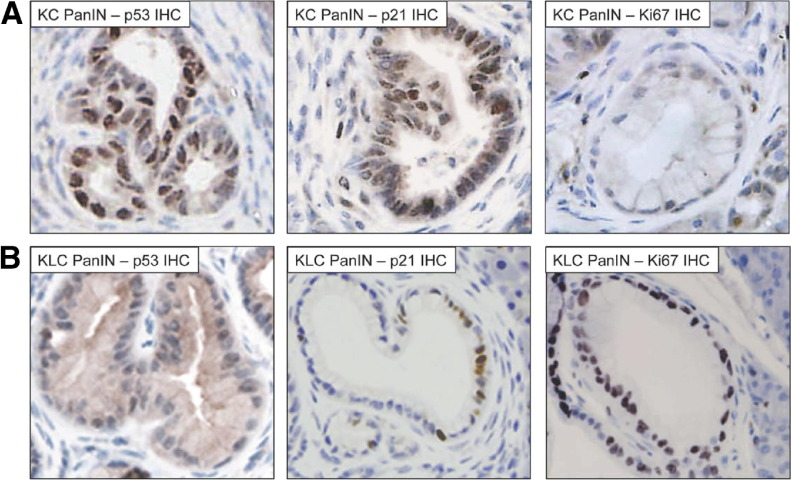
Lkb1 deficiency accelerates Kras^G12D^-mediated pancreatic tumorigenesis through down-regulation of p21. (*A*) Immunohistochemical staining for p53, p21, and Ki67 in pancreatic intraepithelial neoplasia (PanIN) lesions in *Pdx1-Cre Kras*^*G12D/+*^ (KC) mice. (*B*) Immunohistochemical staining for p53, p21, and Ki67 in PanIN lesions in *Pdx1-Cre Kras*^*G12D/+*^*Lkb1*^*flox/+*^ (KLC) mice.

**Figure 4:**
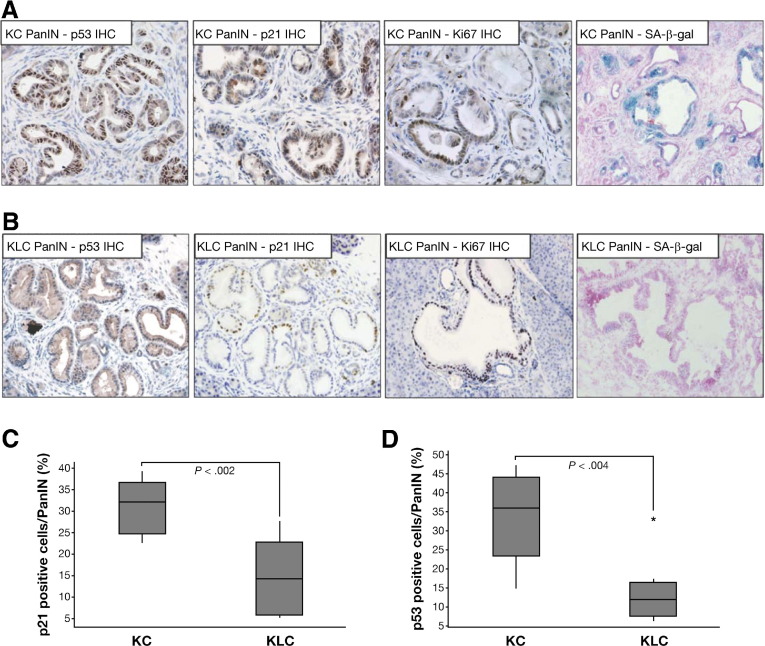
Lkb1 deficiency accelerates Kras^G12D^-mediated pancreatic tumorigenesis through down-regulation of p21. (*A*) Immunohistochemical staining for p53, p21, and Ki67, and senescence-associated β-gal staining in pancreatic intraepithelial neoplasia (PanIN) lesions in *Pdx1-Cre Kras*^*G12D/+*^ (KC) mice. (*B*) Immunohistochemical staining for p53, p21, and Ki67, and senescence-associated β-gal staining in PanIN lesions in *Pdx1-Cre Kras*^*G12D/+*^*Lkb1*^*flox/+*^ (KLC) mice (Supplementary Figure 2 for high-magnification images). (*C*) *Boxplot* showing quantification of p21 staining in PanINs in KC mice compared with KLC mice (n = 6) as indicated (*P* < .002). (*D*) *Boxplot* showing quantification of p53 staining in PanINs in KC mice compared with KLC mice (n = 6) as indicated (*P* < .004).

We sought to further delineate the mechanism by which Lkb1 haploinsufficiency synergizes with activated Kras to promote pancreatic tumorigenesis. Consistent with its in vivo tumor suppressor function, Lkb1 deficiency has been shown to prevent culture-induced senescence.^13^ Re-expression of Lkb1 in cancer cell lines deficient for Lkb1 has also been shown to result in p53-dependent cell-cycle arrest and enhanced expression of p21.^19,20^ On the basis of these results, we wondered whether Lkb1 might act to suppress pancreatic tumorigenesis by promoting growth arrest in vivo through transcriptional activation of p21, because preneoplastic pancreatic lesions in *Elas*-tTA/*tetO-Cre, Kras*^*G12V*^ mice have previously been reported to undergo oncogene-induced senescence, as indicated by positive staining for a number of senescence markers.^29^ We hypothesized that preneoplastic lesions in our *Pdx1-Cre, Kras*^*G12D/+*^*, Lkb1*^*flox/+*^ (KLC) mice would exhibit diminished p21 and p53 expression compared with those lesions found in *Pdx1-Cre, Kras*^*G12D/+*^ (KC) mice. We performed immunohistochemical analysis for both p21 and p53 in PanIN lesions in these mice. High levels of both p21 and p53 were observed in PanINs arising in *Pdx1-Cre, Kras*^*G12D/+*^ (KC) mice (Figure 4*A*,Supplementary Figure 2), compared with normal ducts in these mice, as expected (data not shown). Significantly, however, in PanIN lesions arising in *Pdx1-Cre, Kras*^*G12D/+*^*, Lkb1*^*flox/+*^ (KLC) mice, we observed a considerable reduction in levels of p21 and of p53 (Figure 4*B*, Supplementary Figure 2). We quantified the proportion of cells staining positive for p21 and p53 expression in PanINs from both *Pdx1-Cre, Kras*^*G12D/+*^ (KC) and *Pdx1-Cre, Kras*^*G12D/+*^*, Lkb1*^*flox/+*^ (KLC) mice and confirmed that expression of both was significantly reduced in *Pdx1-Cre, Kras*^*G12D/+*^*, Lkb1*^*flox/+*^ (KLC) PanINs with a median of 14.3% p21-positive cells and 12.0% p53-positive cells, compared with 32.2% and 36.1%, respectively, in *Pdx1-Cre, Kras*^*G12D/+*^ (KC) PanINs (Figure 4*C*, *P* < .002; Figure 4*D*, *P* < .004). Quantitative real-time polymerase chain reaction analysis performed on microdissected tissue demonstrated that transcription of p21 is also decreased in *Pdx1-Cre, Kras*^*G12D/+*^*Lkb1*^*flox/+*^ (KLC) mice, to 0.64% of the levels observed in *Pdx1-Cre, Kras*^*G12D/+*^ (KC) PanINs (data not shown).

**Figure 5:**
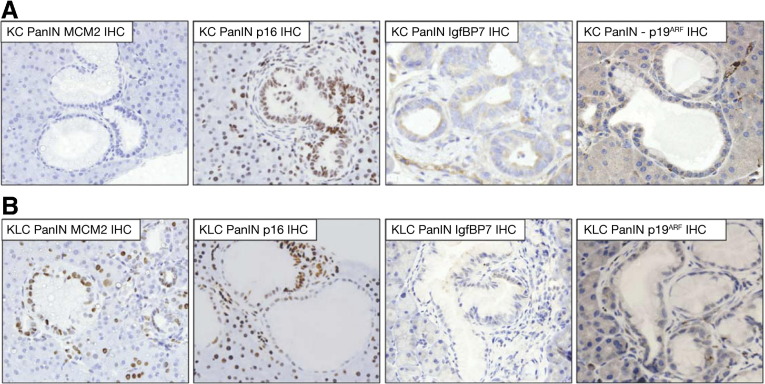
(*A*) Immunohistochemical staining for MCM2, p16, IgfBP7, and p19^ARF^ in pancreatic intraepithelial neoplasia (PanIN) lesions in *Pdx1-Cre Kras*^*G12D/+*^ (KC) mice. (*B*) Immunohistochemical staining for MCM2, p16, IgfBP7, and p19^ARF^ in PanIN lesions in *Pdx1-Cre Kras*^*G12D/+*^*Lkb1*^*flox/+*^ (KLC) mice.

In light of these observations, we also wanted to examine whether preneoplastic lesions in *Pdx1-Cre, Kras*^*G12D/+*^ (KC) mice exhibited signs of growth arrest, implying that Lkb1 deficiency could alter this phenotype through diminished p21 expression. We therefore performed β-galactosidase staining, indicative of senescence (or long-term growth arrest), to address this question. We observed β-galactosidase staining in PanIN lesions and tumors from *Pdx1-Cre, Kras*^*G12D/+*^ (KC) mice (Figure 4*A*), thus confirming previous reports. However, we were unable to detect positive staining in premalignant lesions or tumors in *Pdx1-Cre, Kras*^*G12D/+*^*, Lkb1*^*flox/+*^ (KLC) mice (Figure 4*B*). Conversely, staining for the proliferation marker Ki67 showed that PanIN lesions in 6-week-old *Pdx1-Cre, Kras*^*G12D/+*^*, Lkb1*^*flox/+*^ (KLC) mice were highly proliferative, while lesions in age-matched *Pdx1-Cre, Kras*^*G12D/+*^ (KC) mice exhibited lower levels of staining (Figure 4*A* and *B*). We quantified levels of Ki67 in *Pdx1-Cre, Kras*^*G12D/+*^ (KC) and *Pdx1-Cre, Kras*^*G12D/+*^*, Lkb1*^*flox/+*^ (KLC) PanINs and confirmed that Ki67 expression was elevated significantly in *Pdx1-Cre, Kras*^*G12D/+*^*, Lkb1*^*flox/+*^ (KLC) PanINs, compared with *Pdx1-Cre, Kras*^*G12D/+*^ (KC) PanINs (median % Ki67-positive cells: KC = 10.4, KLC = 79.9; *P* = .0006, data not shown). We performed immunohistochemistry for further markers of senescence/growth arrest, and observed increased expression of p16^Ink4a^ and IgfBP7 and decreased levels of the replication licensing protein MCM2 in *Pdx1-Cre, Kras*^*G12D/+*^ (KC) mice compared with *Pdx1-Cre, Kras*^*G12D/+*^*, Lkb1*^*flox/+*^ (KLC) mice (Figure 5). We did not, however, observe any significant differences in levels of p19^ARF^ (Figure 5, *far-right panels*). These data suggest that Lkb1 acts as a tumor suppressor in the pancreas by inducing p21 expression. Thus, a reduction in Lkb1 levels leads to loss of p21 expression and escape from Ras-induced growth arrest.

### p21 Heterozygosity Accelerates Kras^G12D^-Induced Pancreatic Cancer

**Supplementary Figure 3:**
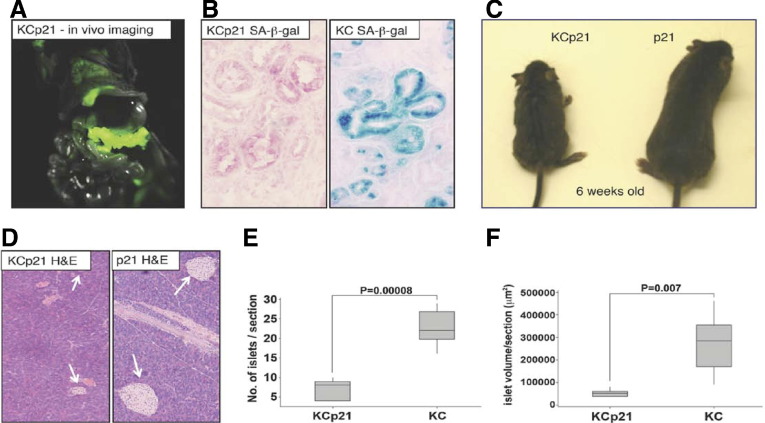
p21 haploinsufficiency synergizes with Kras^G12D^ to induce pancreatic cancer and a dwarfism phenotype. (*A*) In vivo imaging of green fluorescent protein (GFP) fluorescence within the pancreas of a *Pdx1-Cre Kras*^*G12D/+*^*Cdkn1a*^*+/−*^ (KCp21) mouse at 6 weeks old; p21 deficiency allows outgrowth of recombined, and therefore, Kras^G12D^-bearing cells. (*B*) Loss of senescence-associated β-galactosidase staining in pancreatic intraepithelial neoplasia (PanIN) lesions from KCp21 mice, compared with that seen in PanIN lesions arising in *Pdx1-Cre Kras*^*G12D/+*^ (KC) mice. (*C*) Photograph demonstrating dwarfism phenotype in 6-week-old KCp21 mouse compared with a *Pdx1-Cre CdKn1a*/littermate control. (*D*) Representative H&E-stained sections showing reduced islet size in the pancreata of KCp21 mice, compared with *Pdx1-Cre Cdkn1a*^*+/−*^ control mice. (*E*) *Boxplot* showing number of islets per histopathological section of pancreas from KCp21 and KC mice as indicated (*P* = .00008). (*F*) *Boxplot* showing quantification of islet volume per histopathological section of pancreas from KCp21 and KC mice as indicated (*P* = .007).

**Figure 6:**
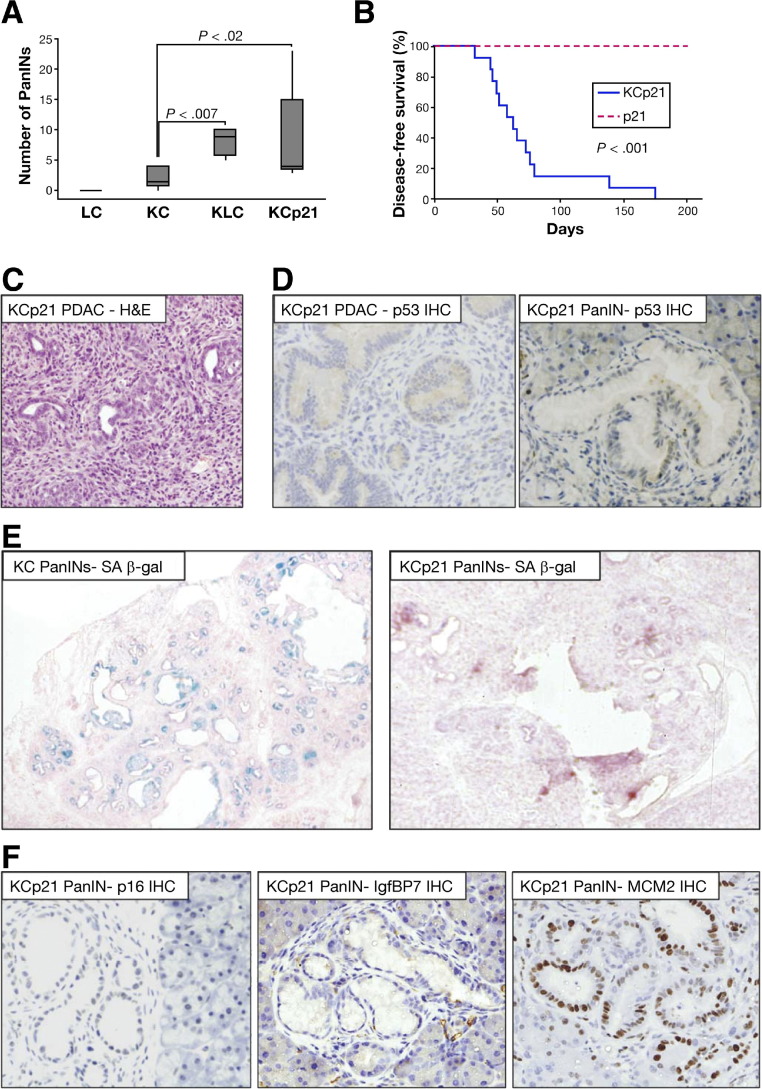
(*A*) *Boxplot* showing number of pancreatic intraepithelial neoplasias (PanINs) per histopathological section of pancreas from wild-type, KC, KLC, and KCp21 mice (n = 6) as indicated. (*B*) Kaplan–Meier survival curve showing disease-free survival of *Pdx1-Cre p21*^*+/−*^ mice (p21, *broken red line*, n = 13), and *Pdx1-Cre Kras*^*G12D/+*^*p21*^*+/−*^ mice (KCp21, *solid blue line*, n = 13). (*C*) H&E-stained section of a pancreatic ductal adenocarcinoma (PDAC) from a KCp21 mouse. (*D*) Immunohistochemical staining for p53 in PDAC and PanIN arising in KCp21 mice. (*E*) Senescence-associated β-gal staining in PanIN lesions from KC and KCp21 mice. (*F*) Immunohistochemical staining for the senescence markers p16, IgfBP7, and MCM2 in PanIN lesions from KCp21 mice.

In order to examine this hypothesis further, we tested whether p21 deficiency could similarly cooperate, in place of Lkb1, with activated Kras to induce pancreatic tumorigenesis. We crossed *p21*^*+/−*^ (*Cdkn1a*^*+/−*^) mice into our model to generate *Pdx1-Cre, Kras*^*G12D/+*^*, p21*^*+/−*^ (KCp21) mice. These mice all developed PanIN lesions by 6 weeks of age, at an incidence similar to that observed in *Pdx1-Cre, Kras*^*G12D/+*^*, Lkb1*^*flox/+*^ (KLC) mice, and at significantly increased incidence compared with *Pdx1-Cre, Kras*^*G12D/+*^ (KC) mice (Figure 6*A*;*P* = .02). These *Pdx1-Cre, Kras*^G12D/+^*, p21*^*+/−*^ (KCp21) mice were also affected by a dwarfism phenotype. Potentially, this was a result of pancreatic islet insufficiency (Supplementary Figure 3), which required that mice be sacrificed early (disease-free survival shown in Figure 6*B*). Importantly though, PanIN lesions in these mice did progress to form PDAC (Figure 6*C*) in 6 of 13 mice, with a median age at sacrifice of 75 days, while the remaining mice exhibited widespread neoplastic changes at time of sacrifice. We did not observe any senescence-associated β-galactosidase staining of PanIN lesions in *Pdx1-Cre, Kras*^*G12D/+*^*, p21*^*+/−*^ (KCp21) mice, in contrast with PanIN lesions in *Pdx1-Cre, Kras*^*G12D/+*^ (KC) mice (Figure 6*E*). Moreover, we observed decreased expression of p16^Ink4a^ and IgfBP7 and increased levels of MCM2 in *Pdx1-Cre, Kras*^*G12D/+*^*p21*^*+/−*^ (KCp21) mice compared with *Pdx1-Cre, Kras*^*G12D/+*^ (KC) mice (Figure 6*F*). These results were analogous to those observed in *Pdx1-Cre, Kras*^*G12D/+*^*, Lkb1*^*flox/+*^ (KLC) mice, but in contrast to the growth arrest/senescence and long latency of tumor development in *Pdx1-Cre, Kras*^*G12D/+*^ (KC) animals. Our findings suggest that loss of a single copy of p21 or of Lkb1 is sufficient to overcome Kras^G12D^-induced growth arrest/senescence in the pancreas.

**Supplementary Figure 4:**
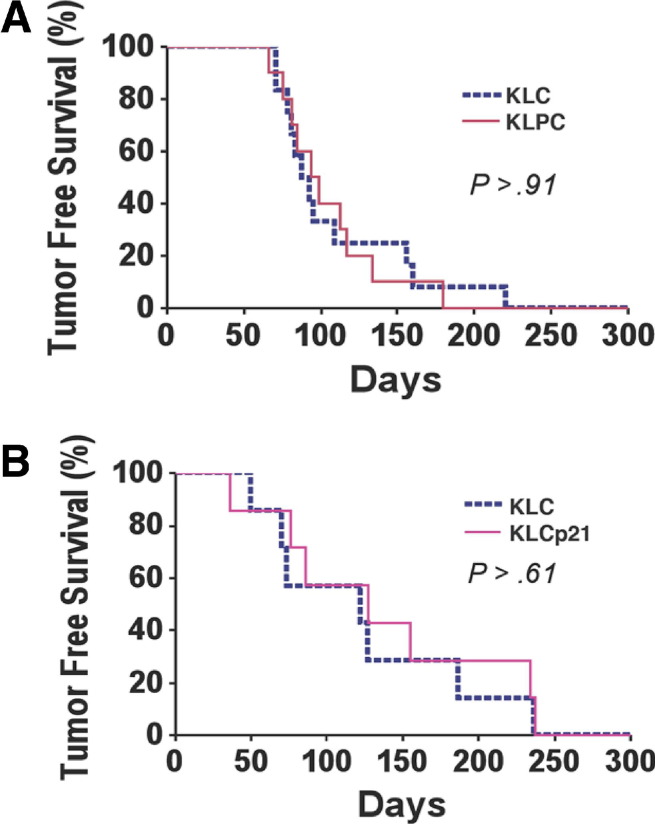
(*A*) Kaplan–Meier survival curve showing tumor-free survival of littermate *Pdx1-Cre Kras*^*G12D/+*^*Lkb1*^*fl/+*^ mice (KLC, *broken blue line*) and *Pdx1-Cre Kras*^*G12D/+*^*Lkb1*^*fl/+*^*Trp53*^*R172H/+*^ mice (KLPC, *solid red line*). (*B*) Kaplan–Meier survival curve showing tumor-free survival of littermate *Pdx1-Cre Kras*^*G12D/+*^*Lkb1*^*fl/+*^ mice (KLC, *broken blue line*) and *Pdx1-Cre Kras*^*G12D/+*^*Lkb1*^*fl/+*^*p21*^*+/−*^ mice (KLCp21, *solid red line*).

We also found that in these *Pdx1-Cre, Kras*^*G12D/+*^*p21*^*+/−*^ (KCp21) animals, p53 was not activated in PanINs, and no accumulation of p53 protein was observed in PDAC (Figure 6*D*), suggestive of the lack of mutation in p53, because p53 mutation frequently results in accumulation of the mutant protein. To further validate this Lkb1-p53-p21 axis in pancreatic tumorigenesis, we crossed *Pdx1-Cre, Kras*^*G12D/+*^*, Lkb1*^*fl/+*^ (KLC) mice with *Pdx1-Cre, Kras*^*G12D/+*^*, p53*^*R172H/+*^ (KPC) mice, and with *Pdx1-Cre, Kras*^*G12D*^*, p21*^*+/−*^ (KCp21) mice to generate triple heterozygous *Pdx1-Cre, Kras*^*G12D/+*^*, Lkb1*^*fl/+*^*, p53*^*R172H/+*^ (KLPC mice), and *Pdx1-Cre, Kras*^*G12D/+*^*, Lkb1*^*fl/+*^*, p21*^*+/−*^ (KLCp21) mice. We observed no acceleration of PDAC formation in either triple mutant mice compared with the corresponding double mutants, indicative of genetic epistasis between Lkb1 deficiency and p53/p21 deficiency (*P* > .91 and .61, respectively, Supplementary Figure 4). These data strongly support our hypothesis that Lkb1 deficiency can substitute for p53 mutation in pancreatic cancer through loss of p21 regulation.

### Decreased Lkb1 Expression in Human PDAC Correlates With Low p21 Expression and Reduced Survival

**Supplementary Figure 5:**
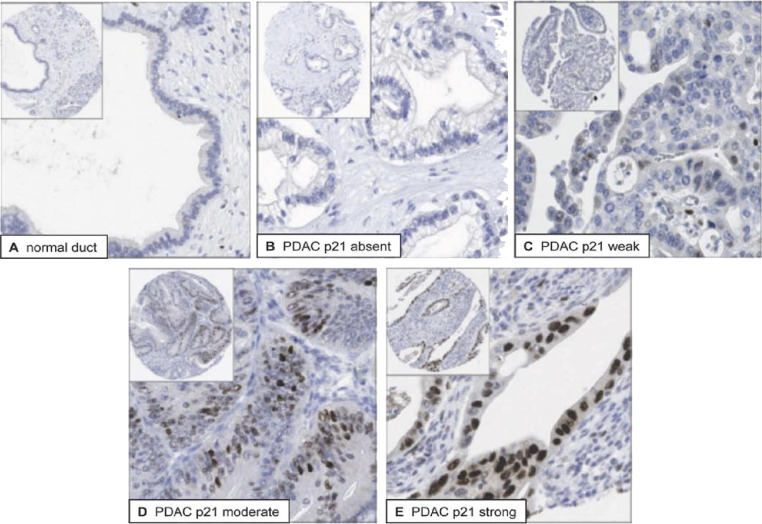
p21 expression in human pancreatic ductal adenocarcinoma. (*A*) p21 immunostaining of normal pancreatic ductal tissue on human tissue microarray (TMA). *(B–E)* Representative immunostaining of p21 in pancreatic ductal adenocarcinoma on human TMA: (*B*) absent, (*C*) weak, *(D)* moderate, and *(E)* strong staining.

**Figure 7:**
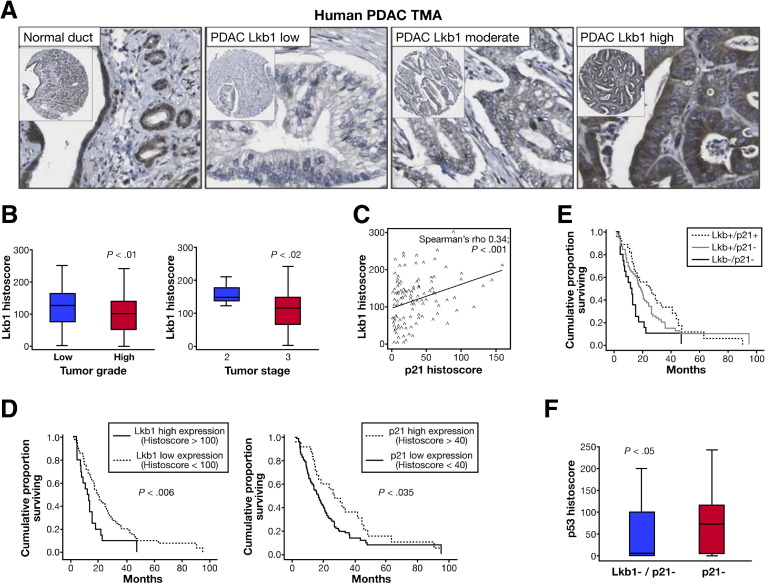
Decreased Lkb1 expression in human pancreatic ductal adenocarcinoma (PDAC) correlates with low p21 expression and reduced survival. (*A*) Lkb1 immunostaining of duct and PDAC on human tissue microarray (TMA). (*B*) Low-grade tumors (n = 81) exhibited a higher level of Lkb1 expression (median histoscore, 128) vs high-grade tumors (n = 33) (median histoscore, 100) (*P* = .01). Stage T2 tumors (n = 13) had a higher level of Lkb1 expression (median histoscore, 150) vs stage T3 tumors (n = 111) (median histoscore, 105) (*P* = .02). (*C*) Correlation of Lkb1 protein with p21 protein expression in 106 cases of PDAC (Spearman's rho correlation coefficient 0.34; *P* < .001). (*D*) Kaplan–Meier analyses showing cases with low Lkb1 expression (n = 20) have poorer outcomes compared to those with high expression (n = 86; *P* = .006), and that cases with p21 low expression (n = 78) have poorer outcomes compared to those with high expression (n = 28; *P* = .035). (*E*) Kaplan–Meier analysis illustrates that Lkb1^hi^/p21^hi^ patients have a more favorable outcome compared to Lkb1^hi^/p21^lo^ and Lkb1^lo^ /p21^lo^ cases. (*F*) *Boxplot* of p53 histoscore in Lkb1^lo^/p21^lo^ tumors (*blue bar*, n = 20) compared with Lkb1^hi^/p21^lo^ tumors (*red bar*, n = 58) (*P* = .05).

We next sought to investigate whether this Lkb1/p21 pathway was relevant to human PDAC development. Lkb1 and p21 immunohistochemistry was performed in a tissue microarray containing 114 cases of primary human PDAC. As expected, we observed Lkb1 staining primarily in the cytoplasm of epithelial cells (Figure 7*A*), while p21 staining was evident in the nuclear compartment (Supplementary Figure 5). Lkb1 staining was present in 98% of stained normal ductal tissue. In PDAC, 19% of cases expressed Lkb1 at a low level (histoscore <100). Expression levels of Lkb1 did not differ in terms of lymph node status or tumor size; however, high tumor grade and stage were significantly associated with lower median Lkb1 expression level (Figure 7*B*; *P* = .01 and *P* = .02, respectively). In univariate analysis, low Lkb1 expression (n = 20) was associated with significantly decreased survival compared with high expression (n = 86) after resection (Figure 7*D*, *left panel*, 11.6 months (95% confidence interval [CI]: 5.7–17.5] vs 19.6 months [95% CI: 13.5–20.6]; *P* = .006). Most importantly, in a multivariate Cox proportional-hazards regression analysis, low Lkb1 expression remained an independent predictor of poor survival, with a hazard ratio of 1.87 (95% CI: 1.09–3.22; *P* = .022).

Given our preclinical data suggesting that low Lkb1 levels caused low levels of p21, we next investigated the expression of p21 on the same human PDAC tissue microarray. Expression levels of p21 were not significantly altered in relation to any clinicopathological parameter; however, low expression of p21 (n = 78) was associated with decreased cumulative survival after surgical resection, compared with high expression (n = 28) (Figure 7*D*, *right panel*, 16.2 months [95% CI: 12.3–20.0] vs 25.7 months [95% CI: 11.7–40.2]; *P* = .035). Strikingly, in these human PDAC cases, Lkb1 expression was demonstrated to correlate directly with p21 expression (Figure 7*C*, Spearman's ρ correlation coefficient 0.34; *P* < .001). Significantly, high expression of both Lkb1 and p21 identified a group of patients with a more favorable outcome and a median survival of 25.7 months (Figure 7*E*, 95% CI: 12.9–40.3). Other predictors of poor survival were higher tumor stage, high histologic grade, larger tumor size, and positive resection margin; however, p21 status did not independently influence outcomes (Supplementary Table 1).

Because the *TP53* tumor suppressor gene is frequently mutated in human pancreatic cancer (40%–70%)^30^ and LKB1 is down-regulated in around 20% of PDAC, we hypothesized that loss of Lkb1-mediated p53/p21 induction might be able to circumvent the need for p53 mutation in human PDAC and thus should not be down-regulated in those tumors with p53 mutation. We therefore investigated levels of p53 accumulation, indicative of p53 mutation, by immunohistochemical staining of the human PDAC tissue microarray. Strikingly, in those tumors that had low levels of LKB1, and, hence, low levels of p21, we did not observe accumulation of mutant p53 (median histoscore = 4.08, n = 20). In contrast, in the subset of tumors that had low p21 with high LKB1 expression, we found significantly higher levels of p53, indicative of accumulation of mutant p53 (median histoscore = 71.3, n = 58, *P* = .05) (Figure 7*F*). In human pancreatic cancer, we have shown that Lkb1 deficiency correlates with loss of p21 expression and with poorer prognosis, and that Lkb1 deficiency may act as an alternative to p53 mutation in human pancreatic tumorigenesis. These results support the hypothesis that Lkb1 acts as a tumor suppressor in the pancreas, and that it functions, at least in part, by inducing p21 expression. Loss of Lkb1 can thus facilitate escape from Ras-induced p21-mediated growth arrest, and promote Ras-induced tumorigenesis in the pancreas.

## Discussion

These data show that Lkb1 haploinsufficiency synergizes with activated Kras in pancreatic tumorigenesis. Mechanistically, we believe this is because of reduced growth arrest/senescence through low levels of p21 in PDAC from these mice. Importantly, our study of human PDAC strongly supports this finding, as low levels of p21 and LKB1 are correlated in human PDACs. Our data are consistent with the previous findings that Lkb1 loss prevents culture-induced cellular senescence,^13^ allows *BRAF* mutant melanoma cells to proliferate,^31^ and cooperates with activating *Kras* mutations in a mouse model of lung cancer.^32^ Indeed, our studies in both the pancreas and intestine suggest strong synergy with Kras signaling, with heterozygosity for *Lkb1* sufficient to drive signaling downstream of Kras.^33^ Overall, these data indicate that levels of Lkb1 are critical in determining the cellular response to Kras activation.

One important question that has been raised through our work and that of others^13^ is whether biallelic mutations in *LKB1* are required for tumorigenesis or whether they may in fact be limiting for tumor progression. Peutz–Jeghers syndrome patients develop benign hamartomas of the gastrointestinal tract and develop intraductal papillary mucinous neoplasm and cystadenomas. Here we have confirmed the previous study of Hezel and colleagues,^27^ who showed that complete loss of Lkb1 in the pancreas leads to formation of benign cystadenomas. Taken together, these data argue that complete loss of Lkb1 leads to formation of benign tumors, that a cooperating oncogenic event is required to drive carcinoma formation, and that the timing of the cooperating oncogenic event may be critical—if it occurs too late the tumor may not progress from a benign state. From the data presented here, we suggest that in sporadic cancer, a single *LKB1* mutation or down-regulation of protein expression would be sufficient to synergize with *KRAS* mutation to drive tumor progression. Analysis of human pancreatic cancers is consistent with this hypothesis; 20 of 106 tumors show a down-regulation of LKB1 compared to normal ductal epithelium and, remarkably, low levels of LKB1 can act as an independent prognostic indicator of poor outcomes of resected pancreatic cancer. In agreement with our findings in the pancreas, when the *LKB1* gene sequence was determined in primary lung adenocarcinomas, only 8 of 27 tumors (of 80 cancers total) that had a mutation or deletion of *LKB1* exhibited biallelic loss,^32^ suggesting that a monoallelic mutation in *LKB1* is sufficient to drive cancer progression. The lack of LKB1 mutations so far observed in human RAS-driven pancreatic tumors may instead be explained by down-regulation at the protein level, or inactivation of the gene by epigenetic means, because hypermethylation of Lkb1 in hamartomatous polyps and in tumors commonly associated with Peutz–Jeghers syndrome has been demonstrated in the absence of mutation of the gene.^34^

We propose that the mechanism for the synergy between *Lkb1* heterozygosity and Kras activation is an escape from Kras^G12D^-induced growth arrest by loss of p53 mediated p21 up-regulation. The reasons for this are multiple, including increased numbers of PanINs in *Pdx1-Cre, Kras*^*G12D/+*^*, Lkb1*^*flox/+*^ (KLC) mice, increased proliferation of PanINs, concomitant reduced expression levels of p53 and p21, reduced expression of senescence-associated β-galactosidase, rapid tumorigenesis in *Pdx1-Cre, Kras*^*G12D/+*^*, p21*^*+/−*^ mice, and the human data showing the correlation between LKB1 and p21 levels. Remarkably, no human tumors that had low Lkb1 expression had high p21 expression. There was a subset of human tumors that had low p21 with high LKB1 expression, presumably because of the fact that multiple different events can cause p21 down-regulation, for example, p53 mutation or TBX2 overexpression.^35^ Indeed, this group of tumors exhibited high levels of p53, indicative of mutant p53 accumulation, suggesting that Lkb1 deficiency can substitute for p53 mutation in human pancreatic tumorigenesis.

Given the plethora of pathways that LKB1 impinges on, it is likely that other pathways may also contribute to the phenotype we see here. However, we failed to see clear up-regulation of phospho−mammalian target of rapamycin within the *Pdx1-Cre, Kras*^*G12D/+*^*, Lkb1*^*flox/+*^ (KLC) PanINs and tumors when compared with the *Pdx1-Cre, Kras*^*G12D/+*^ (KC) PanINs and tumors, although we clearly see reduced levels of the target phospho-AMPK (data not shown). It is possible that within the pancreas, reduced AMPK activation is not sufficient to exert a clear phenotype and indeed heterozygous AMPK knockout mice have no reported phenotype.^36^ Other potential phenotypes of LKB1 deficiency, such as a loss of polarity and differentiation to mucus secretory lineages, could accelerate tumorigenesis in this system.^27,37,38^ However, one of the characteristics of Kras-driven PanINs is an increase in mucin secretion and loss of polarity and, because heterozygosity for *Lkb1* has never been sufficient to drive either of these 2 events, we believe that these are not major contributors to our phenotype, although they may act in synergy with Kras activation.

In conclusion, we have shown that *Lkb1* heterozygosity can accelerate Kras^G12D^-induced PDAC formation. We have observed a marked reduction of p53 and p21 expression in PanIN lesions in these mice compared with mice bearing intact *Lkb1*. This correlation is borne out in human PDAC. We therefore propose that Lkb1 acts as a tumor suppressor in the pancreas through its ability to limit the p53/p21 pathway, thus allowing precursor lesions to more easily overcome the Ras-induced growth-arrest barrier to tumor formation.

## Figures and Tables

**Supplementary Table 1:** Multivariate Cox Regression Analysis of Association of Lkb1, p21 and Clinicopathological Parameters with Overall Survival

	Hazard ratio	95% CI	*P* value
Tumor stage (T3 vs T2)	2.20	1.20−4.41	.014
Tumor size (>30 mm vs ≤30 mm)	1.65	1.09−2.51	.02
Resection margin status (positive vs negative)	1.97	1.16−3.4	.012
Tumor grade (high vs low)	2.54	1.60−4.11	<.001
Vascular invasion (yes vs no)	1.18	0.81−1.72	.38
p21 (Histoscore ≤40 vs >40)	1.09	0.65−1.84	.71
Lkb1 (Histoscore ≤100 vs >100)	1.87	1.09−3.22	.023

CI, confidence interval.

## Supplementary Methods

### Genetically Modified Mice and Animal Care

Animals were kept in conventional animal facilities and monitored daily. Experiments were carried out in compliance with UK Home Office guidelines. Mice were genotyped by polymerase chain reaction analysis. Tumor and metastatic burden was assessed by gross pathology and histology. Animals were sacrificed by cervical dislocation as per institutional guidelines. Organs/tumors were removed and either fixed in 10% buffered formalin overnight at room temperature or snap frozen in liquid nitrogen. Fixed tissues were paraffin-embedded, and 5-μm sections were placed on sialynated/poly-l-lysine slides for immunohistochemical analysis.

### Immunohistochemistry

Formalin-fixed paraffin-embedded sections were deparaffinized and rehydrated by passage through Xylene and a graded alcohol series. Endogenous peroxidase activity was inactivated by treatment with 3% hydrogen peroxide, after which antigen retrieval was performed using microwave-heated antigen unmasking solution (Vector Labs, Burlingame, CA) or by incubation in citrate buffer in a pressure cooker. Sections were blocked in 5% serum for an hour, and then incubated with primary antibody for an hour at room temperature or overnight at 4°C. Primary antibodies used were anti-Lkb1 (Abcam, Cambridge, UK) 1:200, anti-pAMPK (Cell Signaling Technology) 1:50, anti mouse p53 (Vector) 1:100, anti-p21 (Santa Cruz Biotechnologies, Santa Cruz, CA) 1:500, anti-Ki67 (Vector) 1:200, and anti-human p53 (Dako, Carpinteria, CA). Sections were incubated in secondary antibody for an hour (Dako Envision^+^ Kit, or Vectastain ABC system) and the staining was visualized with 3,3′-diaminobenzidine tetrahydrochloride. Alcian blue staining was carried out by incubation in Alcian blue solution (pH 2.5) for 30 minutes, followed by counterstaining in nuclear fast red solution for 5 minutes.

### Reverse-Transcriptase Polymerase Chain Reaction

Polymerase chain reactions were performed on a PTC-200 DNA Engine (Bio–Rad Laboratories, Hercules, CA), using the GoTaq polymerase kit (Promega, Madison, WI) according to manufacturer's instructions. Polymerase chain reaction products were run on a 2% agarose gel, stained with ethidium bromide, and visualized using the GelDoc-It 300 imaging system (UVP, Cambridge, UK). Primers sequences used were: Lkb1 F: GGTCACACTTTACAACATCAC, and R: CTCATACTCCAACATCCCTC.

### Tissue Microarray Analysis

All patients gave written, informed consent for the collection of tissue samples, and the local Research Ethics Committee approved collection. All cases had undergone a standardized pancreaticoduodenectomy. A total of 1500 cores from a total of 224 cases with pancreatico-biliary cancer (including 114 pancreatic ductal adenocarcinomas) with a full spectrum of clinical and pathological features were arrayed in slides. At least 6 tissue cores (0.6 mm diameter) from tumor and 2 from adjacent normal tissue were sampled. Complete follow-up data were available for all cases within the tissue microarray analysis. Lkb1, p21, and p53 expression levels were scored based on staining intensity and area of tumor cells using a weighted histoscore calculated from the sum of (1 × % weak staining) + (2 × % moderate staining) + (3 × % strong staining), providing a semi-quantitative classification of staining intensity. The cutoff for high and low expression of Lkb1 and p21 was a histoscore of 100 and 40, respectively. Statistical correlation between Lkb1 expression and p21 expression in human PDAC was determined by the Spearman Correlation Coefficient analysis. Kaplan–Meier survival analysis was used to analyze the overall survival from the time of surgery. Patients alive at the time of follow-up point were censored. To compare length of survival between curves, a log-rank test was performed. A Cox proportional hazards model was used for univariate analysis to adjust for competing risk factors, and the hazard ratio with 95% CIs was reported as an estimate of the risk of disease-specific death. Variables that were found to be significant on univariate analysis at *P* < .10 were included in multivariate analysis in a backward stepwise fashion. Statistical significance was set at a *P* value of <.05. All statistical analyses were performed using SPSS version 15.0 (SPSS Inc, Chicago, IL).

## Footnotes

Note: To access the supplementary material accompanying this article, visit the online version of *Gastroenterology* at www.gastrojournal.org, and at doi:10.1053/j.gastro.2010.04.055.
