# Corrigendum

**DOI:** 10.1053/j.gastro.2024.05.020

**Published:** 2024-08

Morton JP, Jamieson NB, Karim SA, et al. LKB1 Haploinsufficiency Cooperates With *Kras* to Promote Pancreatic Cancer Through Suppression of p21-Dependent Growth Arrest. Gastroenterology 2010;139:586–597.e6.

During the preparation of the above article, the wrong image was included for IgfBP7 staining of KLC mice in Figure 5B. The authors have amended the figure to include the correct image. The updated Figure 5 is shown below.

**Figure 5:**
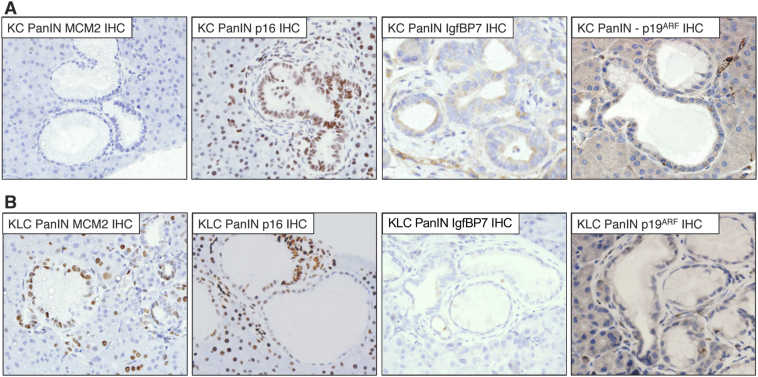

The authors also have provided additional information in the legend for Figure 6E to highlight that a higher magnification image of KC PanIN is shown in Figure 4A.

The correct legend for Figure 6E is: “Senescence-associated β-gal staining in PanIN lesions from KC and KCp21 mice (higher magnification image of KC PanIN is shown in Figure 4A).”

The authors apologize for these errors and state that they do not affect the results described in the figures or the conclusions of the article.

